# Outcome of Fetuses with Diagnosis of Isolated Short Femur in the Second Half of Pregnancy

**DOI:** 10.5402/2012/268218

**Published:** 2012-04-17

**Authors:** José Morales-Roselló, Núria Peralta LLorens

**Affiliations:** Clínica Morales, C/Trafalgar 46 10 2a, 46003 Valencia, 46023 Valencia, Spain

## Abstract

*Objectives*. To assess the outcome of fetuses with isolated short femur detected at 19–41 weeks and determine to what extent this incidental finding should be a cause of concern in fetuses with a normal previous follow-up. *Methods*. 156 fetuses with isolated short femur were compared with a control group of 637 fetuses with normal femur length. FL values were converted into *Z*-scores and classified into 4 groups: control group: *Z*-score over −2, group 1: *Z*-score between −2 and −3, group 2: *Z*-score between −3 and −4, and group 3: *Z*-score below −4. FL values were plotted with the curves representing *Z*-scores −2, −3, and −4. To assess fetal outcome, the frequency of SGA, IUGR, abnormal umbilical Doppler (AUD), Down's syndrome, and skeletal dysplasia was determined for each group after delivery, and the relative risk in comparison with the control group was obtained. Finally, ROC curves were drawn in order to evaluate the FL diagnostic ability for the conditions appearing with increased frequency. *Results*. SGA, IUGR, and AUD were more frequent in the fetuses with short femur. Conversely, none of them presented Down's syndrome or skeletal dysplasia. According to ROC analysis, FL measurement behaved as a good diagnostic test for SGA and IUGR. *Conclusions*. A short femur diagnosis in a fetus with an otherwise normal follow-up determines just a higher risk of being small (SGA or IUGR).

## 1. Introduction

Diagnosis of isolated femoral shortening during the second half of pregnancy has so far been considered a cause for concern as it has been related with Down's syndrome (DS) or skeletal dysplasia (SKD). Although DS can be easily ruled out with amniocentesis, the indication of this technique remains unclear as the procedure risk may overcome in this circumstance the incidence of the disease. Also, knowing if a short femur is the first clue of an SKD is cumbersome, unless pregnancy advances and shortening worsens or any of the accompanying signs is present. In a complete absence of these signs, many of the SKDs are diagnosed after delivery. The aim of this study was therefore to assess the outcome of fetuses with diagnosis of isolated short femur during the second half of pregnancy and determine to what extent anxiety is justified in a low-risk fetal population with uneventful pregnancy controls.

## 2. Patients and Methods

We retrospectively studied 156 fetuses with sonographic diagnosis of isolated short femur (FL *Z*-score below −2 and absence of other morphological anomalies) performed between 19 and 41 weeks of gestation and compared them with a control group of 637 normal fetuses (FL *Z*-score over −2). To avoid biases only one examination per fetus was included in the study and conclusions were based only on relative risks (RRs) with the control group. All fetuses presented an accurate gestational age according to a first trimester crown rump length and underwent a normal and uneventful pregnancy follow-up, including a first trimester DS screening with a combined method (nuchal translucency, *β*-HCG, and PAPP-A) and a midpregnancy anomaly scan. Fetal examination included a complete biometry (BPD, OFD, HC, AC, and FL) and a Doppler assessment of the umbilical artery resistance index.

To avoid gestational influences and make comparisons feasible, FL values were also converted into *Z*-scores according to the following formula: *Z*-score = (value − mean value)/SD and were posteriorly classified in 4 groups: control group (*n* = 637) included fetuses with *Z*-score over −2, group 1 (*n* = 114) included fetuses with *Z*-score between −2 and −3, group 2 (*n* = 27) included fetuses with *Z*-score between −3 and −4, and finally group 3 (*n* = 15) included fetuses with *Z*-score below −4. Raw FL values were afterwards grouped and plotted in a scattergram according to the previous classification, along with three curves representing *Z*-scores −2, −3, and −4.

For the 4 groups described, the frequency of small for gestational age (SGA), intrauterine growth retardation (IUGR), abnormal umbilical Doppler (AUD), DS, and SKD was calculated. SGA and IUGR were considered when the BW was respectively below the 10th and 5th percentile of published BW nomograms [1], and an AUD was diagnosed when the umbilical artery resistance index was over the 95th percentile of published Doppler nomograms [2]. Ultrasound examinations were all performed by the first author with two colour Doppler equipments: a Medison Sonoace 8000 ultrasound machine with a 3–7 MHz convex probe and a Toshiba SSH 140 ultrasound machine with a 3,5 MHz convex probe. Frequencies of SGA, IUGR, AUD, DS, and SKD were compared in order to determine if differences between groups were statistically significant and relative risks (RRs) were calculated with the formula RR = studied group frequency/control group frequency.

Finally, receiver-operating characteristic (ROC) curves were drawn calculating the *P* and the area under the curve (AUC) in order to evaluate the FL diagnostic ability for the diseases appearing with increased frequency. To avoid gestational influence, curves analyzed only FL *Z*-Scores.

Statistical analysis and charts were performed with the software GraphPad Prism 5a, for Apple Macintosh (GraphPad Software Inc, San Diego, CA, USA). Significance was determined using the chi-square test, with a threshold established at *P* < 0.05 (significant) and *P* < 0.001 (highly significant).

## 3. Results

**Figure 1:**
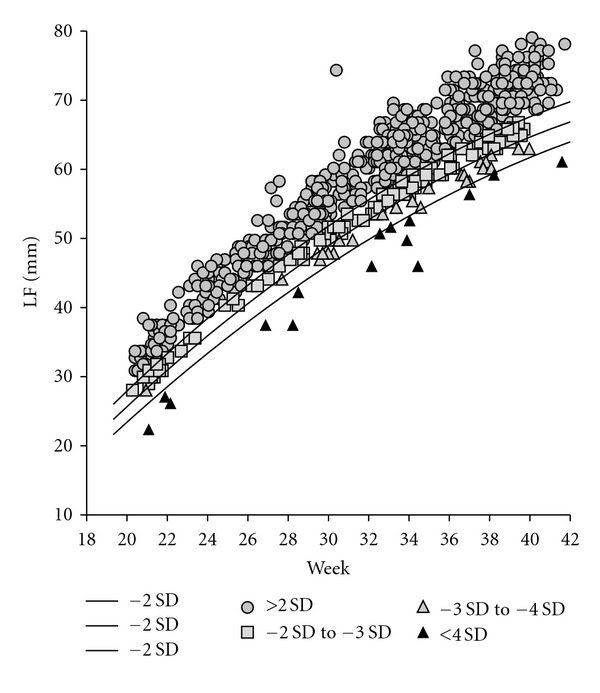
Scattergram of FL values depicted according to the *Z*-score classification. Roundels: fetuses with FL *Z*-score over −2, squares: fetuses with FL *Z*-score between −2 and −3, clear triangles: fetuses with FL *Z*-score between −3 and −4, and dark triangles: fetuses with FL *Z*-score below −4. Curves represent *Z*-scores −2, −3, and −4.

**Figure 2:**
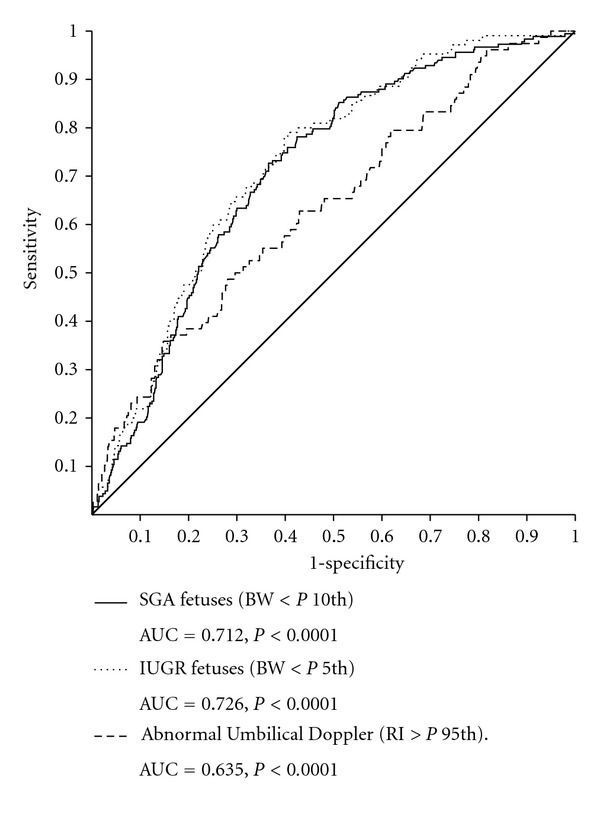
Receiver-operating characteristic (ROC) curves representing the diagnostic ability of the FL *Z*-score for the diagnosis of the three conditions associated with femur shortening (SGA, IUGR, and AUD). AUC: area under the curve.

Results are shown in Figures 1 and 2 and in Table 1.

Figure 1 depicts the scattered values of the 793 FL values, grouped according to the classification earlier described. 637 fetuses (roundels) had a normal FL (*Z*-score over −2), 114 fetuses (squares) had a *Z*-score between −2 and −3, 27 fetuses (clear triangles) had a *Z*-score between −3 and −4, and finally 15 fetuses (dark triangles) had a *Z*-score below −4.

Table 1 shows the distribution frequency and relative risks (RRs) for SGA, IUGR, AUD, DS, and SKD according to the classification in groups described. In the control group (*n* = 637), 122 fetuses (19.1%) were SGA, 63 (9.9%) were IUGR, and 49 (7.7%) had AUD. In group 1 (*n* = 114), 44 fetuses (38.6%) were SGA, 30 (26.3%) were IUGR, and 17 (14.9%) had AUD. In group 2 (*n* = 27), 11 fetuses (40.7%) were SGA, 7 (25.9%) were IUGR, and 7 (25.9%) had AUD. Finally, in group 3 (*n* = 15), 6 fetuses (40.7%) were SGA, 5 (33.3%) were IUGR, and 5 (33.3%) had AUD. No fetuses in group 1, 2, or 3 had a neonatal diagnosis of SKD or DS.

Relative risks for group 1 fetuses were 2 for SGA, 2.6 for IUGR, and 1.9 for AUD. Relative risks for group 2 fetuses were 2.1 for SGA, 2.6 for IUGR, and 3.4 for AUD. Finally, relative risks for group 3 fetuses were 2.1 for SGA, 3.4 for IUGR, and 4.3 for AUD. As no fetus was diagnosed of DS or SKD in any of the 3 groups studied, relative risk for these anomalies was considered to be similar to the general population.

Figure 2 shows the ROC curves and areas under the curve (AUC) for the conditions that appeared with increase of frequency. As only SGA, IUGR, and AUD were found to be associated with FL shortening, only these curves are depicted. To avoid gestational influences, curves analyze only FL *Z*-scores. According to this analysis, the diagnostic ability of FL measurement for SGA and IUGR was high (AUC = 0.726, *P* < 0.0001, and AUC = 0.726, *P* < 0.0001), and the diagnostic ability of FL measurement for AUD was moderate (AUC = 0.635, *P* < 0.0001). The best predictor value for both SGA and IUGR was a LF *Z*-score of −1.03 with a sensitivity of 66% and a specificity of 67%.

## 4. Discussion

A short femur diagnosed in a fetus with normal previous follow-up has represented a cause of concern as it has been related with DS or SKD, serious conditions that cause intrauterine death or severe handicap. In order to clarify if this concern was justified when a short FL was the only finding, we reviewed the published scientific references and analyzed our own database.

Concerning DS, scientific evidence reveals that FL shortening or FL/AC ratio are in fact very soft markers of DS with very low predictive value [3, 4]. Therefore, taking into account the invasive procedure risk, an isolated FL shortening should not be considered an indication for fetal karyotype. On the other hand, SKDs are a wide spectrum of diseases characterized by long bone shortening, sonographically more evident at the femur [5], which tend to show multiple morphological anomalies. SKDs were initially classified according to radiological descriptions. Currently, with the introduction of molecular diagnosis, it has been shown that many of the SKDs derive from mutations within the same gene involved and are therefore considered phenotypic variations of the same disease [6]. The three most common prenatal-onset skeletal dysplasias are osteogenesis imperfecta type 2, thanatophoric displasia, and achondrogenesis type 2, accounting for almost 40% of the cases [7]. As many of these prenatal disorders are lethal conditions, achondroplasia becomes postnatally the most frequent human dwarfism [8].

Published series have shown that SKDs rarely present isolated bone shortening because they are always accompanied by a wide variety of ultrasonographic signs [8, 9]. Therefore, it is important to underline that a fetus with isolated FL shortening will probably not suffer from any type of SKD. The most important accompanying sign of SKDs is the curved femur that appears in more than 40 different conditions [10]. Other signs are the narrow thorax combined with a protuberant abdomen and the anomalies of the skull. Unfortunately these signs are unspecific for the distinct disorders so a precise diagnosis is not always possible [11]. Although the existence of accompanying sonographic signs is always the rule even in cases of extremely rare diseases [12], in a few cases we may find only an isolated short femur. These cases probably represent either benign familiar femur shortening or unilateral isolated femoral hypoplasias, both conditions with good prognosis [13, 14].

Concerning our data, we did not find fetuses with SKD or DS in any of the analyzed groups, even in cases with FL below *Z*-score −4. Conversely SGA was the most frequent anomaly associated with femur shortening, followed by IUGR: 61 fetuses (39%) were SGA (RR = 2.0) and 42 (27%) were IUGR (RR = 2.7). Also 29 fetuses (18%) had an AUD (RR = 2.3). These frequencies were at least 2 times higher than the SGA/IUGR/AUD frequencies in the control group (*P* < 0.001).

We were aware that the frequency of SGA/IUGR/AUD in the control group was also slightly increased, but this could be explained by the high proportion of low class pregnant women attending our clinics. As this circumstance was the same for all the groups studied, we avoided biases taking into account only the RR with the control group. Concerning this RR, we found that a similar result had been reached by Weisz et al. who found that the odds ratio (RR) for SGA in cases of isolated short femur was 3 [15]. In another study Papageorghiou et al. studied 83 cases with isolated femur shortening and showed there were not cases of chromosomal abnormalities or SKD. Conversely early severe IUGR with abnormal umbilical artery Doppler findings and delivery before 37 weeks occurred in 33/83 (40%) cases. These pregnancies also had high rates of pre-eclampsia (36%) and intrauterine death (33%) [16]. Our results, however, are not in agreement with the ones of Todros et al., who found a huge proportion of fetal pathology, including DS and SKD. We were surprised of such incidence, which might be attributed to a selection bias as they were surely studying a selected sample, very different from ours [17].

Femur shortening has been defined by different clinicians according to different thresholds and criteria [11, 16–20]. The most important being the FL/AC ratio below 0.16–0.18, the 10th, 5th, and 3th percentiles and the −3 and −4 SD. However, we consider our results had been the same using any of them as all reflect the same pathological condition. According to our data, fetuses with incidental diagnosis of short femur during the second half of pregnancy, who had undergone a normal pregnancy control, are at risk of being small fetuses (SGA/IUGR) with or without AUD (RR = 2-3). A FL cut-off value of −1 SD allows to detect 66% of small fetuses (SGA and IUGR) with a specificity of 67%.

In summary, the diagnosis of an isolated short femur at 19–41 weeks of pregnancy determines just a higher risk of being small. Concerns about the existence of DS or SKD should be moderated.

## Figures and Tables

**Table 1:** Outcome of fetuses and relative risk according to the femur length *Z*-score.

*N*	**>**2 SD (Control) 637	(Group 1) 114	(Group 2) 27	(Group 3) 15
Outcome				

SGA (183) IUGR (105) AUD (78)	122 (19.1%) 63 (9.9%) 49 (7.7%)	44 (38.6%) 30 (26.3%) 17 (14.9%)	11 (40.7%) 7 (25.9%) 7 (25.9%)	6 (40.0%) 5 (33.3%) 5 (33.3%)

Relative risk				

SGA IUGR AUD	RR = 1 RR = 1 RR = 1	RR = 2.0 RR = 2.6 RR = 1.9	RR = 2.1 RR = 2.6 RR = 3.4	RR = 2.1 RR = 3.4 RR = 4.3

SGA: small for gestational age, IUGR: intrauterine growth restriction, AUD: abnormal umbilical Doppler, DS: Down's syndrome, SKD: skeletal dysplasia, and SD: standard deviation.Number of fetuses with DS or SKD in any group was 0. Relative risk close to 1.
